# A Case of Blastic Plasmacytoid Dendritic Cell Neoplasm Extensively Studied by Flow Cytometry and Immunohistochemistry

**DOI:** 10.1155/2017/4984951

**Published:** 2017-03-20

**Authors:** Martina Pennisi, Clara Cesana, Micol Giulia Cittone, Laura Bandiera, Barbara Scarpati, Valentina Mancini, Silvia Soriani, Silvio Veronese, Mauro Truini, Silvano Rossini, Roberto Cairoli

**Affiliations:** ^1^Division of Hematology, Niguarda Ca' Granda Hospital, Milan, Italy; ^2^Department of Laboratory Medicine, Niguarda Ca' Granda Hospital, Milan, Italy

## Abstract

Blastic plasmacytoid dendritic cell neoplasm (BPDCN) is a rare hematologic malignancy with aggressive clinical course and poor prognosis. Diagnosis is based on detection of CD4^+^ CD56^+^, CD123^high^, TCL-1^+^, and blood dendritic cell antigen-2/CD303^+^ blasts, together with the absence of lineage specific antigens on tumour cells. In this report we present a case of BPDCN presenting with extramedullary and bone marrow involvement, extensively studied by flow cytometry and immunohistochemistry, who achieved complete remission after acute lymphoblastic leukemia like chemotherapy and allogeneic hematopoietic stem cell transplantation.

## 1. Introduction

Blastic plasmacytoid dendritic cell neoplasm (BPDCN) is a rare aggressive hematologic neoplasm, included among acute myeloid leukemia (AML) and related precursor disorders in the 2008 World Health Organization (WHO) classification of hematological diseases and then classified as a distinct entity among myeloid neoplasms in the 2016 revision [1, 2].

Clinical presentation is characterized by an indolent onset of the disease, with extramedullary involvement and tropism towards skin and lymph nodes, followed by systemic dissemination and bone marrow (BM) infiltration [3]. The diagnosis is mainly provided by detection of CD4^+^ CD56^+^, CD123^high^, TCL-1^+^, and blood dendritic cell antigen-2 (BDCA2)/CD303^+^ Lin^−^ blasts [3].

Despite the increasing number of reports and biologic insights about BPDCN, early recognition of the disease still remains a challenge, because its phenotype largely overlaps that displayed by other hematologic malignancies. In this report we present a case of BPDCN, in which extensive flow cytometry (FCM) and immunohistochemistry (IHC) analyses allowed a prompt and accurate diagnosis.

## 2. Case Presentation

**Figure 1:**
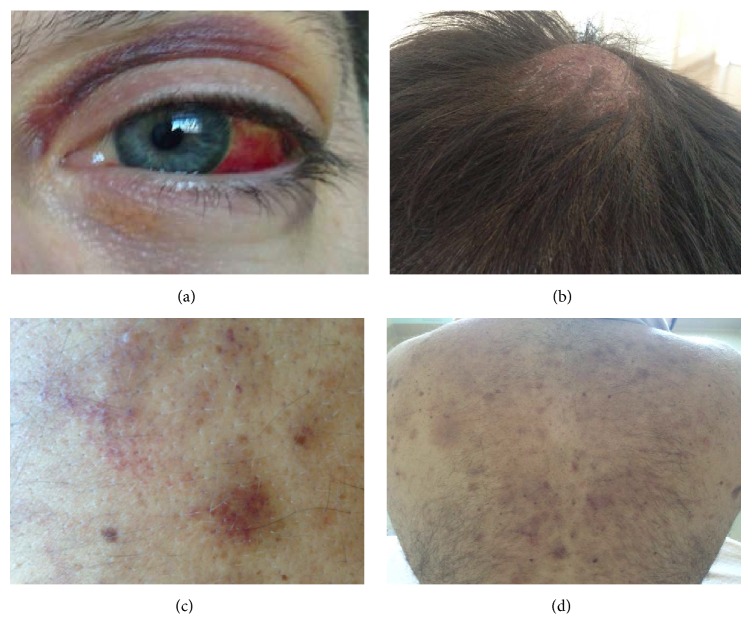
Clinical presentation: close view picture of conjunctival bleeding with eyelid hemorrhage and periorbital edema (a); voluminous infiltrative and erythematous nodule localized on the scalp (b); detailed view of bruise-like brown cutaneous nodules and plaques (c); multiple bruise-like brown cutaneous nodules and plaques localized on the back (d).

A 37-years-old man was referred to our hospital for a two months' history of skin lesions, followed by moderate hearing loss and nosebleed and, lately, by sudden onset of visual impairment and headache. No B symptoms were complained of. Physical exam showed conjunctival bleeding, brown nodular bruise-like lesions on the scalp, neck, and back (Figures 1(a), 1(b), 1(c), and 1(d)), and bilateral cervical and submandibular lymph nodes enlargement. No hepatosplenomegaly was found. At otolaryngology inspection, hypertrophic obstruction of the rhinopharyngeal tract was observed. Ophthalmologic evaluation revealed reduced visual acuity, without retinal or lenses abnormality. Laboratory exams showed white blood cell (WBC) count 6.0 × 10^9^/L (neutrophils 59%, lymphocytes 31%, monocytes 9%, and eosinophils 1%), hemoglobin 14.6 g/dL, and platelet count 92 × 10^9^/L. Blood chemistry and coagulation tests were unremarkable, except for increased lactate dehydrogenase levels (348 U/L, normal < 225 U/L). Anti-DNA/antinuclear antibodies, circulating immune complexes, were absent and serologic tests for hepatitis B and C viruses were negative. Computed tomography scan displayed rhinopharyngeal obstruction by pathological tissue, 2–2.5 cm-sized laterocervical, axillary, abdominal, and inguinal lymph node enlargement, and no cerebral involvement. At positron emission tomography lymph nodes and skin lesions exhibited only a slight fluorodeoxyglucose uptake.

**Figure 2:**
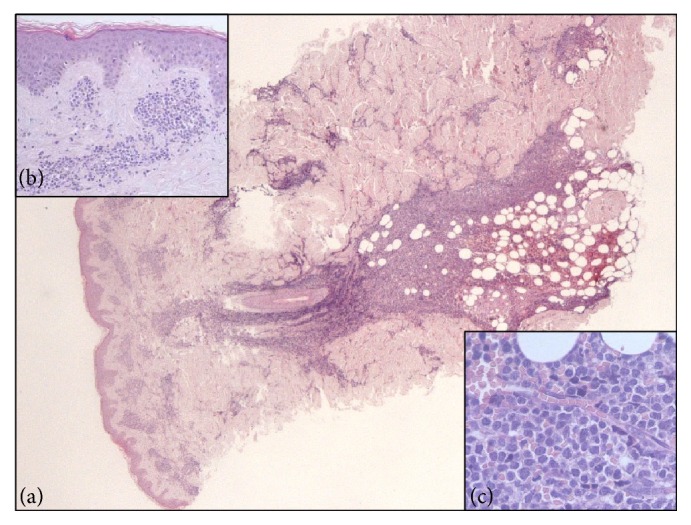
Biopsy of dorsal cutaneous nodule: EE20x dermal-hypodermal blast infiltration, disrupting collagen and muscle tissues, with nodular distribution, sparing epidermidis (a); EE 100x (b) and EE400x (c) dermal-hypodermal monomorphous infiltration by middle-sized blastic elements, with irregular nuclei, small nucleolus, and scant cytoplasm.

**Figure 3:**
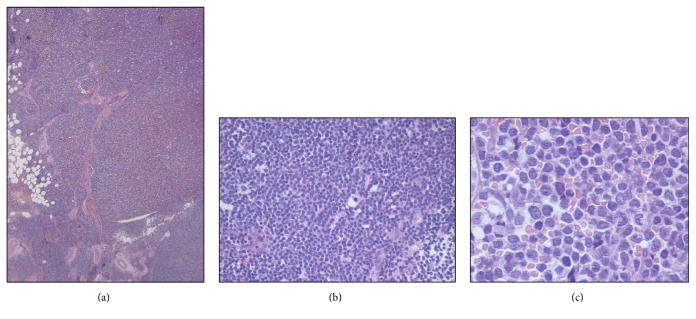
Laterocervical lymph node biopsy: EE20x (a), EE100x (b), and EE400x (c) diffuse and partially nodular infiltration by blast cells with complete effacement of lymph node architecture.

**Figure 4:**
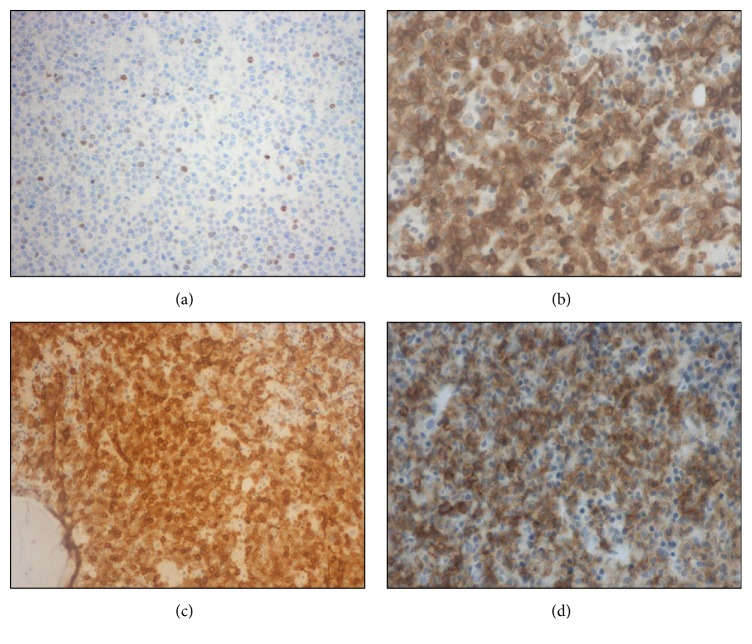
Lymph node immunohistochemistry analysis: immunostaining for TdT showing partial positivity (a); CD4 positive immunostaining (b); CD10 immunostaining on cell surface (c); CD56 positive immunostaining (d).

Skin biopsy showed a diffuse dermal and hypodermal infiltration by immature cells, with irregular nuclei and scant cytoplasm, with a perivascular and periadnexal pattern, involving nervous structures (Figures 2(a), 2(b), and 2(c)). A lymph node biopsy proved complete architectural effacement secondary to massive infiltration by analogous cells with the following IHC expression: CD4^+^ CD10^+^ CD56^+^ CD99^+^ CD123^+^ CD303^+^ TdT^+^ BCL2^+^; CD68PGM1^+/−^ CD7^+/−^ CD43^+/−^ CD2^−/+^; CD3^−^ CD5^−^ CD8^−^ CD20^−^ CD30^−^ CD34^−^ CD79a^−^ CD117^−^ CD138^−^ MPO^−^ TIA1^−^ PAX5^−^ CyclinD^−^ (Figures 3 and 4). Ki67 expression was 80%. T-cell receptor (TCR) gamma chain gene resulted monoclonal.

**Figure 5:**
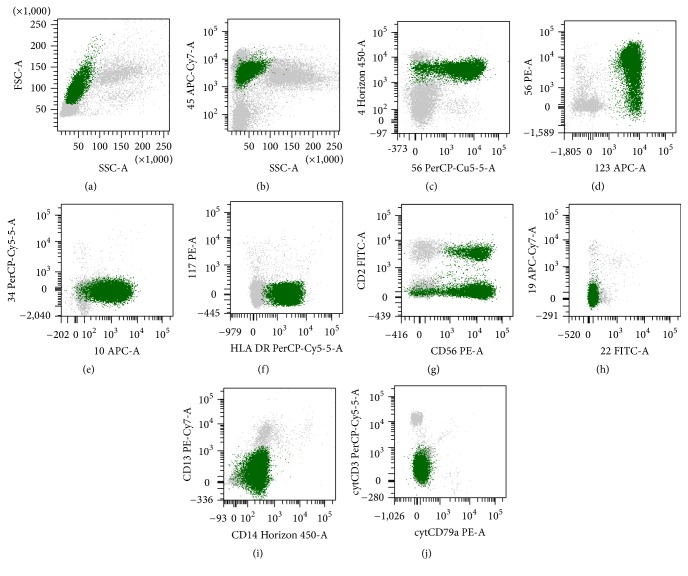
Immunophenotype of the bone marrow showing a large blastic plasmacytoid dendritic cell population (green): the blasts were large-sided cells with variable side scatter properties (a) and were weakly positive for CD45 (b); positive for CD4 (c), CD56 (c, d, e), CD123 (d), CD10 (e), and HLADR (f); partly positive for CD2 (g); and negative for CD34 (e), CD117 (f), CD19 (h), CD22 (h), CD13 (i), CD14 (i), cytCD3 (j), and cytCD79a (j).

Few days after admission, sudden worsening of signs and symptoms and the development of peripheral cytopenias (WBC 2.1 × 10^9^/L, Hb 10.3 g/dL, and platelets 36 × 10^9^/L) were registered. Blood smear revealed the presence of blasts 7%, neutrophils 42%, lymphocytes 44%, and monocytes 7%. BM smear showed 78% middle-size blasts with occasional pseudopodia, characterized by the following antigen expression as detected by FCM: CD45^dim^ CD4^+^ CD10^+^ CD38^+^ CD45RA^+^ CD56^+^ CD123^+^ HLADR^+^; CD2^−/+^ CD7^−/+^ TdT^−/+^; CD1a^−^ CD3^−^ CD5^−^ CD8^−^ CD11b^−^ CD11c^−^ CD13^−^ CD14^−^ CD15^−^ CD16^−^ CD19^−^ CD20^−^ CD22^−^ CD25^−^ CD33^−^ CD34^−^ CD36^−^ CD64^−^ CD66c^−^ CD117^−^ CD138^−^ CD235a^−^ cytCD3^−^ cytCD22^−^ cytCD41^−^ cytCD61^−^ cytCD79a^−^ cytMPO^−^ (Figure 5). Conventional cytogenetics on BM showed normal karyotype, and no BCL2 rearrangement was detected by hybridization and molecular analyses; heavy chain immunoglobulin gene and TCR gamma chain gene showed polyclonal rearrangement. Cerebrospinal fluid FCM displayed a cluster of CD4^+^ CD10^+^ CD56^+^ CD123^+^ blast cells, coherent with occult central nervous system localization.

After an effective steroid debulking, the patient was started on acute lymphoblastic leukemia- (ALL-) like treatment, with three courses of HyperCVAD chemotherapy (fractionated cyclophosphamide, vincristine, Adriamycin, and dexamethasone) and concomitant intrathecal prophylaxis (methotrexate, cytarabine, and methylprednisolone), and achieved complete remission (CR); afterwards he underwent consolidation with allogeneic hematopoietic stem cell transplantation (HSCT) from an unrelated matched donor. At present, thirteen months after diagnosis, he is still in CR.

## 3. Discussion

BPDCN is a very rare and aggressive hematologic malignancy, whose biologic insights and optimal treatment approach are still under investigation.

Different techniques have been employed to address the molecular basis for BPDCN. It has been recognized that the normal counterpart of BPDCN resides in plasmacytoid dendritic cells (pDCs), mononuclear cells produced in the BM and then circulating in blood, lymph nodes, and mucosal sites when an immune response is activated [4, 5]. Great emphasis has been placed on the origin of pDCs. A certain degree of developmental and functional heterogeneity exists within the pDCs population. Indeed, different studies proved evidence that pDCs originate from myeloid precursors, but the possibility of a partial lymphoid contribution to pDCs development has also been postulated [5–7]. Gene expression profiling and sequencing analysis showed that BPDCN shares deregulated genes with both AML and ALL, but it owns a unique molecular signature, distinct from myeloid or lymphoid neoplasms [8]. Conventional cytogenetic analysis shows frequent complex aberrations, often chromosomal losses such as 5q, 12p13, 13q21, 6q23-ter, 9, but no specific karyotypic abnormalities [9, 10]. Several studies identified inactivation of tumour suppressors (RB1, TP53, and CDKN2A), activation of oncogenes (NRAS, KRAS), and mutations in epigenetic regulators (TET2, TET1, DNMT3A, IDH1, and IHD2) that are also frequently mutated in AML and myelodysplastic syndromes; furthermore, mutations in the IKAROS family genes and ATM aberrations have been discovered that are commonly found in lymphoid neoplasms [11–15]. Collectively, these data support that the cell of origin of the tumour can be closer to a myeloid precursor, but a shared trait with lymphoid malignancies cannot be excluded.

Most patients exhibit an indolent onset, with peculiar skin tropism and lymph node involvement, followed by systemic dissemination and BM infiltration. A small percentage of BPDCN is conversely characterized by leukemic presentation at diagnosis [16–21]. Within the spectrum of the disease, different maturation stages of BPDCN have been postulated based on the expression of CD34 and CD117. The double negative subset with high frequency of extramedullary involvement, as in the case of our patient, has been defined as the “mature” one [22]. On the other hand, our patient showed partial TdT positivity. This finding conflicts with a previous BPDCN classification, identifying as more mature the TdT negative cases [23]. Indeed, TdT expression, which is registered in a third of BPDCN, can be regarded as a paramount marker of precursor differentiation [1]. Therefore, the CD34^−^ CD117^−^ TdT+ phenotype of our patient is not appropriately consistent with subgroup definition and suggests the need for further investigation on this matter.

At the screening, the immunophenotype of BPDCN largely overlaps that of other hematologic malignancies, such as AML, extranodal nasal type Natural Killer/T-cell lymphoma, and T-cell leukemia/lymphoma [9, 24]. In our case CD10 positivity was recorded. CD10 is commonly expressed on early, pro-/pre-B cells, but also on T/NK cell precursors, subsequently lost during lymphoid differentiation [25]. Even if commonly reported as a negative antigen, it has been observed in few reports in BPDCN and occasionally in acute myeloid leukemia [18, 26, 27]. Despite the absence of lineage antigen expression, CD10 positivity, together with TdT expression and lymphoid tissues involvement, might have been misleading for diagnosis in the absence of a comprehensive FCM and IHC characterization.

Several reports demonstrate that lymphoid-like chemotherapy is currently the best treatment option for BPDCN, achieving high response rates; the efficacy of ALL protocols might be sustained by deregulation of genes that herald sensitivity to methotrexate, prednisone, and vincristine. However, unlike the majority of lymphoid malignancies, conventional chemotherapy alone does not appear to be sufficient to ensure durable long-term remissions, with an early relapse rate of about 60% of patients achieving CR [17, 21, 28]. As such, our patient demonstrated an excellent response to HyperCVAD chemotherapy that was further consolidated with allogeneic HSCT. In fact, available information derived from retrospective case reports and single-institution experiences suggests that adults may benefit from allogeneic HSCT in first CR, achieving long-term survival with both myeloablative and reduced intensity conditioning regimens [17, 29–31]. Moreover, novel potential therapeutic targets have been identified, such as BCL-2, an antiapoptotic protein commonly overexpressed in BPDCN, as in our case [8, 16, 32, 33]. The chance to find effective targeted therapies furtherly strengthens the need for complete characterization of this neoplasm.
